# Mr. Pym on the Bulam Fever

**Published:** 1816-02

**Authors:** W. Pym


### Medico-Chirurgical Journal and Review.

VOL. I.] FEBRUARY, 1816. [NO. 2.

### PART I.

### ORIGINAL COMMUNICATIONS.

*To the Editors of the Medico-Chirurgical Journal & Review.*

GENTLEMEN,

WITHOUT making any remark upon your partiality or impartiality as Reviewers, I have to thank you for the length of your observations upon Bulam Fever in your Journals for October and November; and as you have left the subject open to me, I shall endeavour to convince you that it is a disease *sui generis;* which point, you say, at p. 331, " you will be forced to concede to me, if I succeed in proving that it is contagious, and that it attacks the human frame but once." Contagion, if I was to enter into it, would lead me far beyond the limits of my present object; and as you have at this moment the work of Sir James Fellowes under review, who is of the same opinion as myself with respect to its contagious powers, I am inclined to drop this part of the subject, in hopes that his evidence will confirm what you appear nearly satisfied with at p. 409 of your Journal, where you say, " We are by no means convinced, however, that the contagion was of foreign origin, but are rather disposed to attribute it to domestic generation during a fever originally non-contagious."

My object then will be to prove that it is a disease, *sui generis, attacking the human frame but once,* trusting that, if I succeed in this last point, you will agree with me that it is contagious and of foreign origin : in support of which I shall bring forward (a gentleman whose name I ought to be cautious in mentioning, as my extract from his anonymous section upon Yellow Fever, in Mr. Johnson's publication, gave so much offence) Dr. M'Arthur, Physician to Deal Hospital; and with respect to him, I cannot help observing, that you were rather too tenacious of your friend's cause. No offence could possibly be intended on my part; and the conclusion drawn by you respecting the extract was certainly wrong, as my object was not to prove any thing against Dr. M'Arthur, but that Dr. Burnett had seen a different disease from that described by him; and if you had quoted the first sentence in the extract, your readers would have been convinced of it.

Dr. M'Arthur, p. 212, says, " Bleeding largely, in the early stage of the fever, has been found of the most eminent service; when employed after the first stage of the fever had passed by, (twenty-four or thirty-six hours) it did injury, and certainly hurried on dissolution." Dr. Burnett bled on the third, and even on the seventh day of the disease; and in the fever which he saw, ninety-five out of a hundred, after venæsection, will show signs of amendment, even after the Bulam fever had time to run its whole course. This is a digression, but an explanation which I have thought necessary, in justification of Dr. M'Arthur as well as of myself.

With respect to the disease being *sui generis*, in my opinion Dr. M'Arthur's description of it goes far to prove it; and if he had happened to have seen the disease in Europe, I make no doubt but he would have been convinced of that as well as of its contagious powers; for in the West Indies, where it was not known that it attacked the human frame but once, the escape of the natives and seasoned Europeans could only be accounted for by the disease being supposed to be non-contagious.

The endemic of Batavia, although in some particulars resembling the fever of the West described by Dr. M'Arthur, certainly differs most essentially in many points, the most prominent of which I shall attempt to distinguish in the following columns;

| *Batavian Endemic.* | *West India Fever.* |
| --- | --- |
| 1st. | 1st. |
| This was attended with distinct paroxysms. P. 171. The length of this paroxysm (the first) varied from six to eight hours, and was generally succeeded by cold rigors. | Dr. M'Arthur, p. 237, says, I have never noticed a remission in the whole course of the fever. |

| *Endemic of Batavia* | *West India Fever.* |
| --- | --- |
| 2d. | 2d. |
| P. 173. Hæmorrhage from the mouth and nose seldom occurred. | P. 236. Hæmorrhage, more or less, takes place from the nose, mouth, &c. and a deposition of blood from the urine. |
| 3d. | 3d. |
| P. 174. The brain appeared to be the organ chiefly affected at first, the stomach and liver in succession. | P. 239. In ten cases of a peculiarly aggravated degree of fever, where much delirium had been present, Dr. M'Arthur opened the head; in five cases, the brain did not exhibit any marked appearance of disease: the stomach, on the contrary, in above a hundred cases which he inspected, shewed the following appearances: |
| | P. 239. Irregular spots, patches and streaks of the internal surface in a state of inflammation, gangrene, or sphacelus; sometimes large portions of the villous coat destroyed. |
| 4th. | 4th. |
| P. 175. No constitution was exempted from the assault of this fever; it seized, with equal or nearly equal violence, on those who had been many years in India, and on the most robust and newly arrived European. | P. 233. The inhabitants of this island, as well as of the other West India islands, are subject to various fevers of the typhoid, catarrhal, or remittent kind; these attack indiscriminately the native or seasoned European, and are as mild as fevers of a similar type in Europe: but the fatal fever, of which I am about to give some account, for the most part, attacks persons from Europe within the first year and half after their arrival in the country, more particularly seamen and soldiers. The natives are not entirely exempt, but to them it rarely proves fatal. |
| 5th. | 5th. |
| P. 181-2. We had the mortification to see our patients, after being rescued from the jaws of | Dr. M'Arthur mentions, that relapses frequently terminate fatally; but from my own experience |

*Endemic of Batavia.*

of death (by the use of mercury), every symptom of fever gone, and after being several days convalescent, with a relish for food, relapse one after another as the soreness left their mouths, and die almost to a man.

*West India Fever.*

rience I cannot call to recollection one instance of it; and if they do occur, they are rare indeed compared to their description in the Endemic of Batavia.

6th.

The convalescence from this, as from all marsh fevers, is very slow. Two of the mildest Cases, 9 and 10, page 205, continued a long time on the convalescent list before they acquired strength.

6th.

Dr. M'Arthur, p. 244. Under this plan, fifty patients out of one hundred, attacked by the genuine endemic fever, will shew evident signs of amendment within the above-mentioned period (24 or 36 hours): from this state they recover with extraordinary rapidity; in one week they are restored to perfect health.

7th.

P. 176. Several of them changed into obstinate intermittents at sea, with great derangement of the liver, spleen, and bowels.

7th.

No change of this kind is mentioned by Dr. M'Arthur, although, at p. 238, several cases of remittent, under his care, terminated in the endemic; neither does he take notice of any derangemant of the abdominal viscera, excepting in thirteen patients, who recovered from black vomiting.

8th.

Page 176. The fatal termination generally happened on the 3d, 5th, 7th, 9th, and not unfrequently the 11th and 13th day; if they passed this period, they generally lingered out twenty or thirty days.

But very few indeed ever ultimately recovered who had slept on shore, and were attacked at that dreadful island Edam.

8th.

Dr. M'Arthur, p. 237. The fever of the Amelia in 1804, and of the Northumberland and Atlas in 1805, terminated fatally from the 2d to the 4th day. The fevers of 1807 and 1808 extended from the 3d to the 5th day.

And as a contrast to the island of Edam, patients recover rapidly from this fever in the West Indies, America, and Spain, and continue their residence upon the same spot where they were taken ill, without danger of relapse or second attack.

*Endemic of Batavia.*

9th.

P. 172. Vomiting of black bilious stuff, resembling the grounds of coffee, frequently commenced early, and continued a most distressing symptom.

In Case 8, p. 201, black vomiting appeared on the fifth day, and continued seven days.

Case 16, page 219, black vomiting came on the 11th day of the disease and continued four days.

Case 7, p. 198, was attacked with black vomiting on the 6th day; after which he landed and walked to the hospital, carrying his hammoc upon his shoulder.

P. 173. The pulse never could be depended on. In the very last stages it has been regular; *but in general it is small, quick, and either hard, or stringy and tremulous;* sometimes, during the reaction of the system, full and hard.

10th.

P. 172. A great proportion changed in a few days to a *bright yellow*, some to a leaden colour; other cases terminated fatally in a very rapid manner too, without the slightest alteration in that respect.

*West India Fever.*

9th.

The third day or stage begins by an amelioration of all the bad symptoms, the vomiting and thirst excepted.

The matter ejected has small membranaceous - looking floculi floating on it, resembling the crust washed from a Port wine bottle; the heat of the skin is reduced; *the pulse sinks to or below its natural standard.*

The patient for an hour or two expresses himself greatly relieved; and at this time, a person not acquainted with the nature of the disease would have hopes of his recovery.

This state, however, is of short duration, and the delusion soon vanishes.

10th.

Yellowness of skin very rarely takes place in this disease, excepting in bad cases.

Dr. M'Arthur mentions in cases where black vomiting was present,

P. 236. Skin of a dirty yellow; parts round the neck and places pressed upon in bed of a livid colour; and, at page 245, (in cases where danger has been apprehended) if the skin and eyes have been yellow, the colour becomes more bright.

From this comparison of the Batavian and West India Endemics, they appear two very different diseases; the black vomiting is the most striking, and the most inexplicable symptom in the Batavian disease; in this last it comes on suddenly, and without the deceitful appearance of convalescence, which takes place in the Bulam fever; and we cannot conceive, that if it was attended, as in the West India fever, with gangrene of the villous coat of the stomach, that the patients could have survived so many hours, as in some cases they did days. Most, if not all of the patients at Edam Hospital, who suffered from black vomiting, took bark and Port wine. Coffee and cocoa are common articles of diet for the sick abroad; and as the surgeon did not remain constantly on shore, or if he did not pay particular attention to the matter discharged, he might have been deceived by the attendants; in support of which, I shall quote Dr. M'Arthur; page 245, he says, "Dark coloured fluids have often been taken for black vomit where the latter did not exist, and thus nurses, and even medical men have been deceived."

This fatal fever, respecting the natives and seasoned Europeans, attacking the newly-arrived sailors and soldiers, and terminating fatally from the second to the fourth day, attended with gangrene or sphacelus of the villous coat of the stomach, is what I call Bulam fever, and which I have proved in my publication did not exist in the English West India islands, or in North America, for a considerable number of years before 1793; and that after the year 1764, it had not visited Spain until 1800 and 1803, when it swept off at least 100,000 of the inhabitants; and that during these two last years, although the disease prevailed to the East and West of Gibraltar, that garrison, (as well as Carthagena, one of the most unhealthy situations in the Peninsula) by the establishment of quarantine regulations, effectually shut out this disease. I have also mentioned that at Gibraltar, during the prevalence of the fever in Spain in the year 1804, when no precautions were used against it, the disease, for the first time in a hundred years, was introduced; and in less than three months, carried off fifty-three officers, a greater number than had died in that garrison during the whole century that it had been under the British Crown. In Barbary, the disease was never known but once; viz. in 1800: since which time that country has escaped its ravages, the Moorish Government evading its belief in predestination, by establishing a strict quarantine upon Spain during the summer months. Philadelphia and New York have also escaped it of late years, by a quarantine against the West Indies.

These observations, together with the established fact of its having prevailed in the most healthy situations, not only in the West Indies, but in Europe, on board ship as well as on shore; I take it for granted are sufficient to prove it to be a disease of foreign origin, and of course contagious. The circumstantial evidence which I shall now adduce, in favor of its attacking the human frame but once, will, I trust, prove beyond a doubt, that it is a disease *sui generis*. And first the following hitherto incredible extract from Sauvage's Nosology, published fifty years ago.

Typhus icterodes *contagiosus est;* albos tantum maxime peregrinos ex regionibus frigidis advenas, Indos, Hybrides, mulatros omnes, exceptis infantibus *una tantum vice afficit;* nigri vero ab eo morbo nunquam afficiuntur.

In my observations upon Bulam fever, page 122, I have mentioned, that all the officers and men quartered in Gibraltar during the prevalence of the disease in 1804, who had had it at a former period in the West Indies, escaped it; and at page 27, that of the whole civil population, amounting to 12000, only 28 persons escaped an attack of it, out of which small number 12 had it at a former period, either at Philadelphia, in Spain, or the West Indies. The same has been confirmed at Gibraltar during the years 1810, 13, 14: there being no well authenticated instance of a second attack; and this fact is so well established in that garrison, that among the quarantine regulations against the introduction of the disease in 1815, *all the troops who have not passed the disease are encamped, while those who have passed it are doing the duty of the town.* At Cadiz, Carthagena, and Malaga, the fact of persons not being liable to a second attack is considered to be as well ascertained as in small pox.

To be more circumstantial, I shall give the following particulars respecting the regiments quartered in Gibraltar during the malady of 1804.

In the corps of Royal Artillery, there were eighteen officers, who were all attacked with the disease, excepting General Smith and Captain Campbell, who had had it in the West Indies.

In the corps of Royal Engineers there was only one officer, viz. Captain Thackery, who had had it in the West Indies, and he was the only officer who escaped it, excepting Colonel Fyers, who kept himself and family in quarantine.

In the 2d or Queen's regiment there were not less than twenty-five officers; five of them, viz. Col. Jones, Major Kingsbury, Captain Walsh, Paymaster Wainwright, and Assistant Surgeon Borlase, had had the fever in the West Indies (ten years before) and all escaped it at Gibraltar, where every other officer in the regiment was attacked by it.

In the 10th regiment, every officer (excepting Capt. Carpenter, who had had it in the West Indies) was attacked by it.

In the 13th regiment, with its full complement of officers, there were eight; viz. Lieutenant Colonel the Honourable C. Colville, Lieutenant Colonel Dana, Lieutenant Colonel Scott, Major Belford, Captain Wilkinson, Captain Brown, Quarter-Master Murray, and the Adjutant, who had had it in the West Indies, and all escaped it at Gibraltar, although every other officer in the regiment was attacked by it.

This same regiment, with six of the above-named West India officers, and ten who had had the fever in Gibraltar, returned to the West Indies in 1809 or 1810, and all escaped the disease, although the regiment suffered severely, and buried nine of the officers newly appointed.

In the 54th regiment, with its full complement of officers, (at least thirty) only three escaped an attack of the fever; viz. Colonel Darby, Captain Louis, and Surgeon O'Dwyer, and they had had it before in the West Indies.

This regiment, three years afterwards, went to the West Indies, filled up with new officers and men; and after being eighteen months in Jamaica, was attacked by and suffered severely from this disease, when all those who had had it at Gibraltar escaped it.—Vide Mr. Redmond's Letter, p. 73—Observations on Bulam Fever.

In the corps of Royal Barrack Artificers, every officer and man was attacked by the disease, excepting Serjeant Jones, who had had it in the West Indies.

In the regiment of Rolle, with its full complement of officers, there was only one, viz. Lieutenant Muller, who had had it in the West Indies, and he was the only officer who escaped it.

The staff of the garrison consisted of General Sir Thos. Trigge, General Drummond, Captain Darling, Captain Thackery, General Barnett, Lord Pelham Clinton, Major Andrews, Captain Parsonage, Captain Bruce, Reverend J. Hughes. The four first had had the fever in the West Indies, and escaped it at Gibraltar; the six last were attacked by it, of which number four fell victims to it.

The Medical men who were at Gibraltar during the first ten weeks of the disease were twenty-four in number; six of them, viz. Bell, Borlase, Lully, O'Dwyer, Franco, and Pym, had had it in the West Indies, and all escaped it; while the remaining eighteen, viz. Dr. Nooth, Lewis,

Benyon, Glasse, Maxton, Patton, Redmond, Rouvier, (Kenning doubtful) Cortes, Jay, M'Guire, Desgerrois, L'Odron, Baynes, Burd, Geddes, Minorcan, were attacked by it, and the seven last fell victims to it.

Two more instances of the Bulam fever not attacking a second time, were in the 70th and 55th regiments; the first suffered severely from fever in the West Indies in the year 1794, and returned to that country from Europe in 1800, filled up with new officers, with the exception of six, viz. Colonel Dunbar, Colonel Elliott, Captain Johnstone, Captains Lawrence, Boat, and Hutchinson, who had had the fever at a former period, and who now escaped it, although the corps lost ten of the newly appointed officers in a very short time, and suffered so severely as to be under the necessity of returning to Europe a mere skeleton. The 55th regiment was stationed in the Island of St. Lucia in 1796, where it was nearly annihilated by the yellow fever; after which, the skeleton of the corps returned to England, was filled up with officers and men, and after being six years in Europe, embarked for Jamaica, where it arrived in 1802. In this island it again suffered severely from the yellow fever, twenty-one of the newly appointed officers having fallen victims to it. Mr. M'Millan, surgeon to the forces (formerly surgeon to the regiment) says, it is worthy of remark, that every individual in the corps was attacked by this fever, excepting eleven, viz. Lt. Col. Hogg, Capt Lee, Brown, Dickson, Jones, Crighton, Humphries, Muttlebury, Carpenter, Wilkins, who had had it at St. Lucia, and himself, who had had it at St. Vincent in 1796.

Upon a moderate computation, there were one hundred and fifty officers at Gibraltar in 1804 who had not had the disease before, and twenty-five who had passed it in the West Indies; and making an allowance for one or two doubtful cases, where the disease was so mild as not to confine the patient to bed, one hundred and forty-five at least out of the hundred and fifty were attacked by it while every individual of the twenty-five who had had it before escaped it.

With my evidence in favour of this wonderful peculiarity, which I consider an object of the greatest consequence to establish, as furnishing us with a barrier of security on the side of the healthy, and of humanity on that of the sick, I shall close my observations upon this very singular and much more fatal disease than the plague; this last, during nine months that it prevailed at Malta, not having destroyed more than 4000 of the inhabitants out of a population of at least 100,000; while the Bulam fever at Gibraltar, out of a population of about 16,000, destroyed 6000 in less than three months.

What I have said is with the view of doing good; I am an enemy to every kind of controversy, and was very slow in taking up the gauntlet thrown down by Drs. Bancroft and Burnett! If any of my observations require explanation, of if any doubts exist in your minds respecting the proofs which I have brought forward, I shall be happy in doing my endeavour to clear them up.

I am, &c.

W. PYM.

